# EVALUATING T-CELL AGE-RELATED IMMUNE PHENOTYPES IN THE CONTEXT OF BIOLOGICAL AGING IN HEALTH AND RETIREMENT STUDY

**DOI:** 10.1093/geroni/igac059.1742

**Published:** 2022-12-20

**Authors:** Ramya Ramasubramanian, Helen Meier, Sithara Vivek, Eric Klopack, Eileen Crimmins, Jessica Faul, Janko Nikolich-Žugich, Bharat Thyagarajan

**Affiliations:** University of Minnesota Twin Cities, Minneapolis, Minnesota, United States; University of Michigan, Ann Arbor, Michigan, United States; University of Minnesota Twin Cities, MINNEAPOLIS, Minnesota, United States; University of Southern California, Leonard Davis School of Gerontology, Los Angeles, California, United States; University of Southern California, Los Angeles, California, United States; University of Michigan, Ann Arbor, Michigan, United States; University of Arizona, Tucson, Arizona, United States; University of Minnesota Twin Cities, MINNEAPOLIS, Minnesota, United States

## Abstract

Cellular changes in the adaptive immune system accompanies the aging process and contributes to an age-related immune phenotype (ARIP) characterized by decrease in naïve T (TN) cells and increase in memory T (TM) cells. However, a population level marker of ARIP associated with biological aging and age-related chronic conditions has not been evaluated previously. We developed two ARIP measures based on well understood age related changes in T cell distribution: TN/ (TCM (Central Memory) + TEM (Effector Memory) + TEFF (Effector)) or TN/ TM in CD4+ and CD8+ T cells. We compared them with more commonly used ARIP measures such as CD4/CD8 ratio and CD8+ TN cells by evaluating associations with chronological age and phenotypic age using linear regression and association with multimorbidity using multinomial logistic regression. CD8+ TN and TN/TM had the strongest inverse association with chronological age (beta estimates: -3.41, -3.61; p-value< 0.0001). CD4+ TN/TM had the strongest inverse association with phenotypic age (beta estimate = -0.74; p-value < 0.0001) after adjustment for age, sex, race and CMV serostatus. CD4+ TN/TM was inversely associated with co-occurring chronic conditions (odds ratio for 2 conditions and 3 conditions vs. 0 conditions: 0.74 (95%CI: 0.63-0.86) and 0.75 (95% CI: 0.63-0.90), respectively) after adjustment for age, sex, race, CMV serostatus, smoking, and BMI. CD4+ TN/TM had a stronger association with phenotypic age and age-related morbidity compared to other ARIP measures. Future longitudinal studies can help us evaluate if CD4+ TN/TM can predict risk of aging related outcomes.

